# Social media in medicine: a personal perspective about opportunities, challenges, and best practice

**DOI:** 10.46989/001c.125931

**Published:** 2024-11-23

**Authors:** Mohamad Mohty, Bipin Savani

**Affiliations:** 1 Sorbonne Université; 2 Vanderbilt University

**Keywords:** social media, medical education, academic collaboration

In today’s digital age, social media has become a transformative force, significantly impacting the field of medicine. While traditionally known for face-to-face consultations and academic discussions, healthcare professionals are increasingly turning to platforms like X (formerly known as Twitter), Facebook, or LinkedIn (to mention just a few) to share knowledge, collaborate, and even enhance patient care. This editorial explores the growing role of social media in medicine, highlighting its advantages, inconveniences, and practical tips for effective use from a personal perspective.

## Why do we need social media in medicine?

The advent of social media has dramatically changed the way communication occurs in the medical field. Social media is no longer just a tool for personal use but a crucial platform for academic collaborations, patient care, and educational outreach. The ability to connect with peers across the globe allows for a level of camaraderie and support previously unattainable. Importantly, social media helps answer queries quickly and advance projects faster than conventional communication methods. In addition to speed, social media offers a “freedom” of communication—an environment where stakeholders can share experiences and knowledge without the constraints of geographical boundaries. This virtual network fosters education, awareness, and the dissemination of information, making it an invaluable tool in modern healthcare. Another advantage is the environmentally friendly aspect, reducing the need to travel to meetings and office consumables.

## The inconveniences of social media in medicine

However, while the benefits of social media are clear, there are also notable drawbacks. One of the main inconveniences is the immediate and often fragmented nature of communication. Here, one can raise a critical question: does social media actually improve communication, productivity, and collaboration? The promptness of interactions can sometimes lead to misunderstandings, as the brevity of posts and messages does not always allow for nuanced or detailed clear explanations. Additionally, social media tends to decrease face-to-face interactions, potentially eroding the depth of professional relationships. The platform’s emphasis on short, fragmented statements can also hinder creativity and make it challenging for professionals to engage in deep, focused work. Multitasking and frequent interruptions are common side effects, often reducing productivity rather than enhancing it.

## Best practices for using social media in medicine

Given these challenges, it is essential for healthcare professionals to approach social media with a set of best practices that balance its advantages and disadvantages. One key tip is to manage expectations—both for oneself and for others. While social media can be a powerful tool, excessive use does not necessarily equate to improved productivity or better communication. It is important to maintain a balance and avoid becoming overwhelmed by the constant stream of information. Users are advised to completely disconnect from time to time. Like other forms of addiction, taking breaks from social media can lead to withdrawal symptoms, such as anxiety or the feeling of “missing out.” However, over time, disconnection can result in a sense of clarity, focus, and refreshment. This strategy is particularly helpful in avoiding burnout, a common issue among healthcare professionals.

Another crucial piece of advice is to treat people with respect online. Social media is not the place to make careless remarks, which can be hurtful or misunderstood. Additionally, it is important to maintain professionalism and confidentiality, particularly when discussing sensitive medical information. Informal exchanges can be beneficial, but they should never compromise the ethical standards of the medical profession.

## Navigating the risks of social media

Despite best efforts, things can still go wrong. Thus, it is vital to remember that not all followers are friends—some may use the platform to spread negativity or engage in hateful behavior. Blocking spammers and establishing clear boundaries is essential for maintaining a safe and professional online environment. Moreover, healthcare professionals should avoid sharing personal issues on social media, as these platforms are not private, and information can be accessed indefinitely. Establishing and adhering to personal rules for social media use can help ensure that online activity remains productive and beneficial.

## Conclusion

In conclusion, while social media offers significant advantages in terms of communication, collaboration, and knowledge sharing in the medical field, it also comes with certain risks and challenges. By managing expectations, setting clear boundaries, and maintaining professionalism, healthcare professionals can leverage social media as a powerful tool without letting it negatively impact their personal or professional lives. With thoughtful use (**Table 1**), social media can continue to enhance the way the medical community interacts, learns, and grows in this increasingly digital world.

**Table 1.** Recommendations for managing your reputation and navigating social media.

**Establish a professional presence**	Create official accounts	Set up dedicated professional accounts on platforms like LinkedIn, X (Twitter), and Facebook. Use these accounts solely for professional interactions.
	Use a professional photo	Choose a high-quality, professional profile picture that conveys approachability and expertise.
**Define «your brand»**	Identify your niche	Determine your areas of expertise and the audience you want to reach (patients, colleagues, students, etc.)
	Consistent messaging	Ensure your posts reflect your professional values, expertise, and the type of content you want to be associated with.
**Content strategy**	Share knowledge	Post informative content related to your field, such as articles, research findings, or health tips.
	Engage with current events	Share your insights on relevant health news or medical events, providing thoughtful commentary.
	Educational content	Create or share videos, infographics, and articles that educate patients about medical conditions and treatments.
**Patient interaction**	Respect privacy	Never disclose patient information or personal stories without consent.
	Be responsive	Engage with comments and messages in a timely manner. Address concerns or questions professionally, but avoid offering specific medical advice online.
**Monitor your online presence**	Set up alerts	Use Google alerts or similar services to monitor mentions of your name or practice.
	Regularly check your accounts	Review your social media profiles and online reputation periodically to address any misinformation or negative comments.
**Crisis management**	Prepare for negative feedback	Develop a strategy for handling non-constructive criticism or negative reviews. Respond professionally and consider addressing legitimate concerns privately.
	Stay calm and professional	If faced with public criticism, remain calm and composed. A measured response can enhance your reputation.
**Network and collaborate**	Connect with peers	Engage with other professionals in your field. Networking can lead to collaborative opportunities and increase your visibility.
	Join professional groups or organizations	Participate in online discussions and events hosted by professional medical associations
**Privacy settings**	Adjust privacy settings	Ensure your personal accounts have strict privacy settings to keep your personal life separate from your professional presence
	Be cautious with personal content	Avoid posting personal opinions that could be controversial or misinterpreted in a professional context
**Stay informed**	Follow trends	Keep up with the latest trends in social media and how they can impact healthcare communication
	Participate in training	Attend workshops or webinars on social media use for healthcare professionals to enhance your skills and knowledge
**Ethical considerations**	Adhere to your own code of conduct	Ensure that your conduct aligns with clear guidelines and professional standards
	Be authentic	While professionalism is key, being genuine and relatable can help build trust with your audience

### AUTHORSHIP

Conceptualization: Mohamad Mohty (Lead) and Bipin Savani. Writing – original draft: Mohamad Mohty (Lead). Writing – review & editing: Mohamad Mohty (Lead) and Bipin Savani.

### ETHICAL APPROVAL

Not applicable.

### CONSENT TO PARTICIPATE/INFORMED CONSENT

Not applicable.

### CONSENT FOR PUBLICATION

Not applicable.

